# Ensuring Inclusion of Adolescent Key Populations at Higher Risk of HIV Exposure: Recommendations for Conducting Biological Behavioral Surveillance Surveys

**DOI:** 10.2196/publichealth.7459

**Published:** 2017-06-20

**Authors:** Lisa Grazina Johnston, Justine Sass, Jeffry Acaba, Wing-Sie Cheng, Shirley Mark Prabhu

**Affiliations:** ^1^ Independent Consultant, UNICEF, East Asia and Pacific Regional Office Valencia Spain; ^2^ UNESCO Section of Education for Inclusion and Gender Equality Paris France; ^3^ Youth Lead Bangkok Thailand; ^4^ UNICEF East Asia and Pacific Regional Office Bangkok Thailand

**Keywords:** adolescents, sex workers, sexual minorities, drug users, ethics, sampling

## Abstract

Ending acquired immune deficiency syndrome (AIDS) depends on greater efforts to reduce new human immunodeficiency virus (HIV) infections and prevent AIDS-related deaths among key populations at highest HIV risk, including males who have sex with males, sex workers, and people who inject drugs. Although adolescent key populations (AKP) are disproportionately affected by HIV, they have been largely ignored in HIV biological behavioral surveillance survey (BBSS) activities to date. This paper reviews current ethical and sampling challenges and provides suggestions to ensure AKP are included in surveillance activities, with the aim being to enhance evidence-informed, strategic, and targeted funding allocations and programs toward ending AIDS among AKP. HIV BBSS, conducted every few years worldwide among adult key populations, provide information on HIV and other infections’ prevalence, HIV testing, risk behaviors, program coverage, and when at least three of these surveys are conducted, trend data with which to evaluate progress. We provide suggestions and recommendations on how to make the case to ethical review boards to involve AKP in surveillance while assuring that AKP are properly protected. We also describe two widely used probability sampling methods, time location sampling and respondent driven sampling, and offer considerations of feature modifications when sampling AKP. Effectively responding to AKP’s HIV and sexual risks requires the inclusion of AKP in HIV BBSS activities. The implementation of strategies to overcome barriers to including AKP in HIV BBSS will result in more effective and targeted prevention and intervention programs directly suited to the needs of AKP.

## Introduction

Ending human immunodeficiency virus (HIV) or acquired immune deficiency syndrome (AIDS) relies on greater efforts to reduce new HIV infections and prevent AIDS-related deaths among key populations at highest risk. Whereas data are limited, studies from low and concentrated epidemic countries suggest that HIV prevalence is disproportionately high among adolescents, aged 10-19 years, who sell sex, engage in same-sex relationships, and inject drugs [1]. Ignoring that these behaviors occur among adolescents places them at even higher risk of HIV infection and creates barriers to HIV testing, and, when needed, essential care and treatment [2]. Adolescents under the age of 18 years who sell sex and are likely to be defined as sexually exploited under human rights law [3,4], tend to be the most ignored and least protected group due to controversy and lack of clarity on how to meet their needs [5-7]. In addition, adolescent key populations (AKP) including adolescent males who have sex with males (MSM); male, female, and transgender adolescents who sell sex; and adolescents who inject drugs face numerous vulnerabilities including low education; low access to health care; and high levels of stigma, discrimination, and violence [2]. A punishing legal environment and severe taboos around same-sex relations, selling sex, and injecting drug use tend to drive these behaviors underground, reinforcing the exclusion of adolescents and perpetuating the infection cycle [2,8].

Since early 2000, HIV biological behavioral surveillance surveys (BBSS) have been a key component of national responses to HIV [9,10]. BBSS, conducted every few years worldwide among adult key populations, is the routine collection of strategic information to measure HIV and other infections and biological and behavioral trends; to model the HIV epidemic; to determine allocation of limited resources and funding; to enhance efforts to more effectively respond to local, national, and global HIV prevention strategies; and to measure program coverage. For countries that have conducted at least three BBSS, trends provide information about different countries’ progress in responding to HIV. Many questions in the BBSS are sensitive, including those related to sexual behaviors; drug use; HIV testing history; visits to sexually transmitted infections services; and personal experiences of being arrested, raped, stigmatized, and discriminated against. Unfortunately, many of these surveillance activities do not collect data from persons under the age of 18 years. Some surveys have managed to collect data from participants as young as 15 years [11,12]; however, these data are not disaggregated (by age and sex), and the sample sizes comprising 15 to 19 year olds are often not sufficient for meaningful analysis [13,14]. To fill this gap, some countries have conducted separate studies on younger cohorts; however, many studies use non-probability sampling methods from which inferences about the population cannot be made [15,16].

The lack of continuous, systematic collection, analysis, and interpretation of data on AKP has resulted in a dearth of strategic information needed for the planning, implementation, and evaluation of essential HIV programs. This paper discusses the ethical barriers related to sampling AKP, and suggestions on how to overcome them, and presents recommendations on how to include AKP in BBSS using time location sampling (TLS) and respondent driven sampling (RDS).

## Methods

### Ethical Barriers to Sampling Adolescent Key Populations (AKP)

One of the barriers to AKP inclusion in BBSS is ethical constraints. In almost all countries, adolescents under 18 years are considered children and entitled to the protection of their rights under the Convention on Rights of the Child [17]. Compared with adults, children are more vulnerable to exploitation, abuse, violence, and other harmful outcomes of research, and therefore, require additional safeguards [18-20]. In order for children to participate in research, informed consent is usually required from both the child and the child’s parent or guardian. The failure of institutional review boards to approve self-consent and waive guardian permission in conducting HIV surveys is a significant barrier to AKP participation. AKP may fear being stigmatized, punished, or in some cases victimized by their families if guardian permission results in the disclosure of their behaviors, sexual orientation or gender identity, or HIV status [21]. As a result, surveys may end up with smaller samples and unrepresentative findings. Convincing governments to approve self or proxy (eg, trained social worker serving as a proxy guardian) consent may require building consensus of the value of AKP inclusion and promoting their right to participate, whereas at the same time ensuring their protection from harm. Key ethical parameters of data collection involving key populations under the age of 18 years include (1) informed consent; (2) domestic laws governing child protection; (3) identification of, and referral to, services for AKP; and (4) biological testing. Within each of these parameters, considerations and suggestions for supporting the inclusion of AKP in BBSS are presented in Table 1.

**Table 1:** Ethical considerations and suggestions for conducting biological behavioral surveillance surveys (BBSS) among adolescent key populations (AKP) under the age of 18 years.

Topic	Considerations and suggestions
Obtaining informed consent for studies involving adolescents	Meet with and receive input from all relevant groups and agencies when designing protocols and consent forms, and when considering who will need consent from parents or guardians. For instance, in some countries (1) the age of majority may be younger than 18 years, and (2) children who are married, living independently, or in child-headed households may be considered “liberated minors” and thus, may not require additional consent from a parent or guardian.
	When requesting consent for surveys on sensitive issues from a parent or guardian on behalf of AKP^a^, it may be necessary to keep the nature of the survey vague (ie, refer to a survey on sexual risk or drug use as a “health” survey) and list sex or drugs as one of many health issues being assessed.
Domestic laws governing adolescent protection	Review domestic laws for mandatory reporting of disclosures of adolescent abuse, neglect, violence, or exploitation; consider how mandatory reporting would affect the final research outcome (eg, Will AKP refuse to participate if their information is not confidential? and Will AKP refuse to report certain types of information?) and decide whether a waiver is needed.
	Discuss issues of including or waiving mandatory reporting with adolescent protection officials, social workers, rights advocates, partner agencies, etc.
	Approval of such waivers may only be possible through the national ethical review board or a senior-level adolescent protection authority.
Identification of, and referral to, services for adolescents	Identify and develop a comprehensive list of potential services for AKP (ie, services able to address issues of violence, abuse, neglect or exploitation, and other services such as drop-in centers, shelters, helplines, government or non-government social welfare, vocational training, school programs, health care, legal aid) to respond to AKP special situations.
	Provide referral lists to AKP who participate in a study, whether or not they disclose harm or high-risk behavior.
	Be able to identify which specific service an adolescent may need to access (ie, do not have a list only containing referrals for those who need sexual abuse counseling or detoxification from drugs).
	Ensure that research protocols include clear procedures for making discreet, direct referrals to service providers, should AKP request such assistance. Unless there are mandatory reporting requirements, confidentiality must be respected.
	Work with qualified adolescent protection or health professionals to establish guidelines and meet with potential service providers to assess the capacity, expertise, and resources to respond to direct referrals, including how to respond to urgent or acute abuse, neglect, violence, or exploitation cases.
	If no services exist in the survey area or staff is not equipped to respond to referrals from AKP participants, consider budgeting for and establishing a temporary team of trained service providers to whom AKP can be referred during and shortly following data collection.
	Consider providing transportation arrangements so that service providers in nearby areas can meet with AKP who request support. Plan these arrangements in cooperation with protection and health professionals.
	Develop plans to determine how to accommodate AKP needing long-term or specialized support beyond the research.
Eligibility	Many labels used by researchers to describe key populations (ie, people who inject drugs, male or female sex workers) may not be recognized by AKP engaging in the same behaviors. Refer to the behavior guiding the eligibility, rather than the population group.
Support during the survey	Have available a professionally trained social worker or advocate (a person or service provider with qualifications to provide information and support to an adolescent in distress).
Biological testing	When considering inclusion of a biological component, find out (1) if the country’s ethical standards allow testing on adolescents. If so, what are the laws governing HIV^b^ or other infections testing of adolescents? (2) How is pre- and post-test counseling conducted for and test results provided to AKP and/or parents and guardians? and (3) Whether there are available and appropriate referrals for care, management, and treatment for AKP with positive test results.

^a^AKP: adolescent key populations.

^b^HIV: human immunodeficiency virus.

Informed consent for AKP under the age of 18 years should include considerations beyond general ethical assurances included in any protocol. Extra effort may be needed to ensure that adolescents understand all of the elements in a consent process, including the purpose of the research, the kinds of information to be collected, how confidentiality will be maintained, the interview procedure (in particular that the participant does not have to answer questions with which she or he does not feel comfortable, and that the interview can be stopped at any time), and a contact number for more information about the study or to make a complaint [22]. For instance, in the Philippines, AKP were required to understand and repeat back 4 key items of consent in order to participate in a BBSS using RDS and TLS: (1) participation is voluntary, (2) information is confidential (no one will know what you tell me), (3) participation involves an interview and HIV counseling and testing, and (4) participation will help improve services for adolescents [23]. Another safeguard has been to have on-site social or health workers as “parental proxies” to provide consent on behalf of or in addition to AKP under the age of 18 years [24].

Some countries have specific regulations that disclosures of violence, abuse, neglect, or exploitation of a child override confidentiality and must be reported to relevant authorities. Government employees or particular professions (eg, social workers, health workers, and teachers) or any person aware of an incident must report it. If there are no exemptions for mandatory reporting for the research, then a waiver from an appropriate authority is needed so that interviewers are not required to report abuse disclosures without the adolescent’s approval. It is essential for survey planners to (1) review domestic mandatory reporting laws of disclosures of child abuse, neglect, violence, or exploitation and consider how reporting would affect the final research outcome; and (2) discuss options to waive mandatory reporting by adolescent protection officials, social workers, rights advocates, and partner agencies.

Collecting biological specimens from AKP under the age of 18 years will normally require informed consent from parents and guardians. In many situations, parents and guardians will have access to the test results [25], which can be a strong impediment to AKP getting tested, especially given the sensitivity of an HIV result and the implications of sexual activity or injecting drug use [2]. In light of the increased risk of HIV infection faced by adolescents, some countries are reevaluating and adapting their current legal and policy constraints requiring parental consent for adolescents wanting confidential HIV counseling and testing [21,26,27]. Twelve countries in Asia and the Pacific now have HIV testing laws in place that allow people under 18 years to consent independently to HIV testing [2].

### Methods to Sample Adolescent Key Population (AKP)

Much of our knowledge about adolescent health comes from household- and school-based surveillance, both of which rely on populations that have sampling frames [28]. However, these surveys typically miss populations at higher risk for HIV exposure, many of whom have unstable living environments and housing and prefer to remain “hidden” from law enforcement and other government authorities. Furthermore, these studies fail to capture strategic information on HIV, including HIV and other infections prevalence, program coverage, or specific risk behaviors. There are currently 2 probability-based sampling methods that have been used successfully to sample adult key populations without sampling frames for BBSS—TLS (also known as venue-day-time sampling) and RDS [10,29,30]. Both of these methods allow participants to remain anonymous (ie, no need to collect name, address, or other personal details that can be used to contact a participant) and are therefore, effective at sampling populations that practice risky behaviors and/or face stigma and discrimination. Knowledge about the target population is needed before deciding on which methodology to use. For instance, TLS requires that AKP congregate in identifiable and accessible locations such as street corners, markets, transportation centers, clubs, bars, or other areas [9,31,32], and RDS requires that the population be socially networked so that AKP can identify and recruit other eligible AKP.

TLS was first used to estimate HIV seroprevalence among young MSM (15-22 years) in the United States [33]. The method entails identifying days and times when populations congregate at specific locations (ie, brothels, city blocks, bars, and so on), constructing a sampling frame of time and location units, randomly selecting and visiting time and location units (the primary sampling units), and systematically intercepting and collecting information from consenting individuals. The number of persons at each location provides a sampling weight that can be used a priori, to draw a self-weighting sample, or post priori, in analysis. Data collection takes place at the venue, if space (or venue owner) permits, or in a mobile site near the location, such as a van, or by making appointments to come to a designated study site. The major contribution of TLS over other cluster sampling methods is the ability to account for the fact that key populations are not statically associated with a particular location and often move between multiple locations during a single day. As such, TLS allows researchers to construct a sample with known properties, make statistical inference to the larger population of location visitors, and theorize about the introduction of biases that may limit generalization of results to the target population.

RDS was first used to sample people who inject drugs in the United States [34]. Briefly, RDS begins with a handful of non-randomly selected participants (referred to as seeds) who enroll in the survey and upon completion, receive recruitment coupons that they use to recruit their peers [34-37]. Participants recruited by seeds make up the first wave of participants, who in turn recruit the second wave of participants. This recruitment process continues until the sample comprises multiple recruitment waves and ends once the desired sample size is reached. RDS provides a primary incentive for completing an interview and for recruiting peers. The use of uniquely numbered coupons identifies who recruited whom, while maintaining anonymity. Someone receiving a coupon can decide whether to enroll or not. The major contribution of RDS over other chain referral sampling methods is the ability to harness information about people’s social networks to make inferences about the population. Data on who recruited whom are used to adjust for differential recruitment, and the measurement of each participant’s social network size (the number of peers they know who also know them) is used to weigh the data to control for the overestimation of those with larger social networks and the underestimation of those with smaller social networks.

Both methods can, and have been, used for HIV and non-HIV related surveys of AKP (10-19 years) [38,39]. For example, in Asia, TLS was used in a survey conducted among MSM, male sex workers, and transgender persons (15-24 years; 30.6% of which were between the ages of 15-19 years) in Chiang Mai and Phuket, Thailand (n=827) [40]. In this study, using oral testing for HIV, 13.1% of AKP were HIV seropositive [40]. In Cambodia, TLS was used to sample 1204 males and 1166 females (10-24 years; 52.4% of which were boys and 53.2% of which were girls between the ages of 15-19 years), who were unmarried, and considered high risk based on their visibility at high risk venues. The sample comprised 252 hotspots (ie, bars, night clubs, karaoke parlors, massage parlors, street corners, football fields, snooker clubs, and computer game shops) in the capital city and 7 other provinces [41]. Although information about HIV-related risk behaviors was captured, no biological testing was performed, and there is no indication of adjustments in the analysis for sizes of venues and frequency of visits. Another survey using TLS that directly targeted young key populations living on the streets in the Russian Federation and Ukraine, mapped metro and train stations, street markets, feeding centers or fast-food sites, parks, and computer clubs [42]. This survey used 2 mobile teams in vans in which participants consented to participate, were interviewed, and underwent pre- and post-test counseling and a rapid HIV test.

RDS was used in several provinces of Thailand among young (15-24 years) females who exchange sex for money and goods, MSM, young non-Thai migrants, and transgender youth [43]. Although RDS recruitment worked well, the sample sizes were not sufficient enough to capture many adolescents, and although information about HIV-related risk behaviors and testing was captured, no biological testing was performed. These limitations were also found in RDS surveys conducted among young MSM in Monywa and Yangon, Myanmar [44].

Web-based RDS has been developed to sample hard to reach populations through messaging and emails [45-47]. Although this method does not allow for the collection of biological information since there is no face-to-face contact between research staff and participants, it can be useful and efficient for collecting behavioral information from AKP. For instance, in China, an RDS survey is being planned for adolescent males who have sex with males using a Web-based application. A working group of young MSM and representatives from community-based networks are involved in the questionnaire and survey design and will help in the selection of seeds. Although the inclusion of young key populations, most of whom do not have research skills, involves additional time, their input into the questionnaire has resulted in the prioritization of interview questions, the inclusion of adolescent appropriate language, and important local terms.

When using TLS and RDS to conduct research on AKP, it is important to consider how the methodology should be modified in order for it to be accepted by, and appropriate for, adolescents. When selecting time periods for sampling AKP, one TLS survey of adolescents who use drugs adjusted their data collection activities to avoid hours when activities such as school, work, or chores were most likely to occur. When selecting a data collection site, an RDS survey of street youth in Albania anticipated that waiting space would be needed for older siblings, parents, or other caretakers accompanying an adolescent participant or for younger siblings who were in the care of an adolescent participant [39]. An extra staff person was hired to engage younger siblings with toys, puzzles, games, and other activities as they waited for their adolescent caretaker to finish the survey process. In place of cash incentives, surveys of adolescents have used food items, soap, clothing, games, and other items [39]. Surveys of AKP conducted in Thailand and Myanmar used phone credit as incentives [43,44]. When asking AKP about social network sizes, some RDS surveys have used pictures and counting techniques to help adolescents think about size differentials. For instance, one survey used several different pictures of circles of increasing sizes to help adolescents visualize their most accurate social network size [39]—the smallest circle indicated the smallest social network size (ie, one or two), and the largest size indicated the largest network size (ie, up to 100). Tables 2 and 3 provide an overview of the features specific to each of the sampling methodologies, how each feature is generally implemented, and considerations on how they can be modified for AKP.

**Table 2:** Time location sampling (TLS) methodology features, description of those features, and considerations for applying those features when conducting surveys of adolescent key populations (AKP).

Feature	Current implementation of features	Considerations of features when sampling AKP^a^
Mapping	TLS^b^ requires a complete mapping of all venues in a sampling location.	Venues mapped for adults may not be the same as those mapped for AKP. Involve AKP and groups working with AKP to help map venues. Enumerating AKP at venues may result in some errors since it may be difficult to accurately know someone’s age without speaking to them. Consider using AKP as enumerators during mapping as they may be best able to recognize who are adolescents.
Sampling times and periods	TLS requires that sampling times be randomly selected.	Make sure to include sampling times that conform to AKP’s availability.
Recruitment	TLS involves researchers approaching participants. Sometimes this involves intercepting participants in the street or at a venue and then escort them to another location for an interview and/or testing.	Involve youth as interviewers to minimize intimidation of AKP interviewees. If taking AKP to another area for interviewing or testing, allow for AKP participants to be joined by a friend or trusted person. Make sure the person leading the participant to another location does not appear threatening to either the participant or the public.

^a^AKP: adolescent key populations.

^b^TLS: time location sampling.

**Table 3:** Respondent driven sampling (RDS) methodology features, description of those features, and considerations for applying those features when conducting surveys of adolescent key populations (AKP).

Feature	Current implementation of features	Considerations of features when sampling AKP^a^
Pre-survey research	Conduct pre-survey research to assess populations’ social networks, RDS^b^ acceptability, and logistics.	Organizations working with AKP can help identify sites where AKP spend time, meet friends, find new sex partners, or buy or sell drugs. Sites may or may not be the same as those frequented by adult key populations.
Seeds	Seeds are the initial participants that start the recruitment from within the network of interest.	In surveys of adolescents and adults together, select an ample number of AKP seeds who are more likely to recruit other AKP. In surveys of AKP only, special considerations may be needed to diversify seeds. Some adult diversifications, such as marital status or occupation, may not be applicable to AKP. Consider other diversity factors such as whether they live at home or not, are sexually active or not, in school or not, able to read and write or not, etc.
Incentives	A nominal incentive, usually in the form of local currency, is usually provided to participants who complete the survey process and for recruiting their peers.	Make sure a country’s research ethical standards allow incentives to be provided to adolescents. Incentives used for adults may not be appropriate for adolescents. Involve organizations working with AKP to select appropriate incentives. Ensure that incentives do not induce participation (eg, if it were not for the incentive provided, the participant would not enroll in a survey or would not withdraw from the survey early, given his or her better judgment).
Measuring social network size	For RDS analysis, the number of eligible people that each subject “knows” and has seen during a specified period of time (eg, 2 weeks) is needed. This question is open-ended and gaining accurate responses can be difficult, especially from someone uncomfortable with counting.	Use special probing techniques to assist with and encourage accurate reporting of social network sizes. Some adolescents may need help with coming up with a number.
Interview sites	RDS usually requires recruits to be present at an interview site to undergo the survey.	Interview sites should be easy to access, safe, and comfortable for AKP. In surveys of adolescents and adults together, consider special hours for AKP to enroll when adult participants are not present. Avoid interview sites located close to schools, homes, police stations, prisons, sex work solicitation areas, high crime areas, etc. Consider what to do with family members who bring AKP to participate in a survey. Consider the use of WebRDS [45-47], whereby peers recruit their peers via messaging and email.
Staffing	RDS surveys usually have numerous staff members, including someone to screen for eligibility, interviewers, pre- and post-test HIV^c^ counselors, coupon managers, and so on.	Ensure staff members are trained to interact with adolescents and can recognize signs of distress and able to respond appropriately. Retain a social worker familiar with AKP at the survey site to respond to difficult situations. Ensure staff members understand the consent form and are trained to acquire informed consent from adolescents and/or their parent or guardian without coercing the adolescent. Have a staff member to engage adolescents with activities while they are waiting for an interview. Support staff who may be emotionally impacted by their exposure to adolescents who sell sex, use drugs, have experienced violence, are living with HIV, or have other risks and vulnerabilities.
Coupons	Coupons are used by participants to recruit peers. Coupons convey important information about the survey location, operation hours, etc.	Use simple coupon designs and words that are easily understandable to adolescents. Use pictures in place of words or other creative means to convey important coupon information. If using WebRDS [45-47], coupons can be sent through Web-based applications.

^a^AKP: adolescent key populations.

^b^RDS: respondent driven sampling.

^c^HIV: human immunodeficiency virus.

Both TLS and RDS require additional considerations when collecting biological and sensitive behavioral data from AKP (Table 4). Pre-survey assessments, data collection forms, interviewing, biological specimen procedures techniques, and eligibility descriptions may need to be modified when sampling youth populations. Additional staff may be needed to address the specific needs of AKP, especially those who have been in abusive situations. For instance, in some RDS surveys of adolescents, trained social workers were hired to respond to any participant needing assistance, feeling distress as a result of answering sensitive questions, or expressing that they were in a harmful or abusive situation [23,39]. Once data are analyzed, important findings should be shared with the population and validated. For instance, in the BBSS RDS surveys conducted in the Philippines, the surprising finding that a large percentage of adolescent female sex workers and males who have sex with males were attending school resulted in a validation process [48]. Qualitative research conducted by the Department of Health in schools indicated that even though HIV education was part of the curriculum, few students received adequate HIV information in school. Identified reasons included that teachers were not receiving formal HIV training; that textbooks had outdated HIV information; and that some teachers were not comfortable discussing HIV and skipped lessons about HIV. This exercise resulted in plans to strengthen HIV education, thereby, reaching school-enrolled AKP and building upon HIV BBSS findings.

**Table 4:** Special considerations and suggestions when adapting biological behavioral surveillance surveys (BBSS) among adolescent key populations (AKP) in time location sampling (TLS) and respondent driven sampling (RDS).

Topic	Considerations and suggestions
Pre-survey assessment	An AKP^a^ specific pre-survey assessment may include (1) meetings with and involvement from parents, guardians, or community gatekeepers to ensure AKP participate in surveys, (2) community meetings (without disclosing the full nature of the research that could result in further stigma or reprisals) to garner community acceptance of collecting information from AKP, (3) meetings with government officials, child advocates, and NGOs^b^ working with adolescents to be fully aware of country laws and guidelines governing the involvement of children in research, and (4) research to determine if adolescent-friendly communication technologies (ie, mobile phone apps and Internet websites) or eliciting questions through cell phones and computers can enhance sampling (Note: Web-based surveys do not allow for biological data collection).
Questionnaires and other data collection forms	Materials for adolescents of higher age ranges (eg, 15-19 years) need to be adapted for those of lower age ranges (eg, 10-14 years).
	Keep questionnaires and materials short and simple, and include adolescent-appropriate language.
	Forms to be read by participants should be easy to read using language commonly used by adolescents. Some AKP are not able or do not want to read something—have the option that materials can be read to them.
	Consider the use of drawings to convey important information.
Interviews	Interviews among AKP should be short.
	Allow respondents to take breaks during an interview.
HIV and other testing	Minimize the invasiveness of HIV testing^c^ and other testing procedures. Consider using oral swabs or finger pricks instead of venous blood.
Eligibility	Labels used by researchers to describe AKP (ie, people who inject drugs, male or female sex workers) may not be recognized by adolescents engaging in the same behaviors. Refer to the behavior, for example, selling sex in exchange for money, rather than the population group (sex workers).
Support during the survey	Have available a trained social worker or child advocate (a person or service provider with qualifications to provide information or support).
Dissemination and validation	Once data are analyzed, go back to AKP to disseminate and validate data.

^a^AKP: adolescent key populations.

^b^NGO: non-governmental organization.

^c^HIV: human immunodeficiency virus.

## Discussion

This paper is a product of an international meeting convened to share lessons learned, improvements and innovations, and outputs on HIV surveillance activities. This was the third such meeting (2004 in Addis Ababa, Ethiopia; 2009 and 2015 in Bangkok, Thailand) but the first to include a focus on AKP, reflecting recent concerns about their absence in HIV BBSS [49]. Data from surveys conducted among AKP thus far have provided essential information to respond to the specific needs of AKP, including overlapping risks (ie, adolescents who inject drugs and sell sex), sexual behaviors, barriers to service uptake, violence, as well as HIV- and non-HIV-related (ie, reproductive and mental) health needs [14,48,50].

We note that there are several ethical barriers to sampling adolescents; however, using the strategies proposed here, AKP under the age of 18 years can be involved in research and still be protected from undue harm. Countries enacting laws allowing adolescents to access HIV testing without parental consent may serve as leverage to argue the benefits of conducting research among AKP without parental consent, as long as there are sufficient ethical protections and access to needed care and treatment.

During the past decade or more, HIV prevalence and associated risk behaviors among adult key populations worldwide have been successfully measured using innovative sampling methods including TLS and RDS. However, it is unclear why the lessons learned from surveys of adult key populations are not being harnessed to capture strategic information about AKP in existing BBSS or in surveys targeting AKP. In some cases, more time and expense may be needed to capture AKP. Given that survey designs need to be tailored to reach AKP, they comprise a small part of the key populations and may be more hidden than their adult counterparts. Many surveys of adult key populations include adolescents as young as 15 years [11,12]; however, disaggregated analysis that might inform donors and governments about 15-19 year olds, including disaggregation by sex, is rarely presented [14]. Other important information including the age of initiation of drug use or sexual behavior from surveys of adult key populations have been useful for advocating new policies to benefit AKP.

As in most surveys of adult key populations, research among AKP should include the involvement of the research population in the survey planning, implementation, analysis, and dissemination. Researchers should endeavor to provide avenues to involve adolescents, especially AKP, in all phases of research as such involvement will increase their ownership of the data collected and a higher likelihood that the findings are used on their behalf to develop more useful national HIV testing, treatment, care, and support strategies. When designing research, adolescents may have insight into current behavior trends such as which drugs are being used and which venues are being frequented by AKP, and which tools and language will be most effective. In addition, older adolescents can be included as part of the data collection and analysis team. Future directions for sampling AKP could include developing novel data collection strategies such as using game interfaces with colors, sounds, and levels of earning points when responding to questionnaires in an effort to keep adolescents engaged. As more BBSS’ are conducted among AKP using TLS and RDS, it is hoped that additional lessons learned will be shared to ensure the most optimal implementation and adaptation of these methods to sample these underserved high risk populations.

### Conclusions

Despite the fact that deaths due to AIDS continue to increase for adolescents, while they have decreased for all other age groups since 2010 [13], little data are available to ensure that adolescents are provided adequate prevention, intervention, care, and treatment services. Although there are some ethical barriers to conducting surveillance among AKP, with effort and collective action, many of these can be overcome. There are currently effective methods for collecting and using data from surveys of adult key populations that could easily be used in AKP. At a minimum, existing data of adolescents in adult BBSS should be disaggregated into adolescent age groupings. It is necessary to take the extra time and resources to effectively collect meaningful data that will provide needed strategies to address the health needs of AKP.

## Abbreviations

AIDS: acquired immune deficiency syndrome
AKP: adolescent key populations
BBSS: biological behavioral surveillance surveys
HIV: human immunodeficiency virus
MSM: males who have sex with males
RDS: respondent driven sampling
TLS: time location sampling
